# The Paediatric BCG Vaccine Century: From Historical Success to Future Innovations

**DOI:** 10.1111/apa.70182

**Published:** 2025-06-23

**Authors:** Stefan H. E. Kaufmann

**Affiliations:** ^1^ Max Planck Institute for Infection Biology Berlin Germany; ^2^ Max Planck Institute for Multidisciplinary Sciences Göttingen Germany; ^3^ Charité—Universitätsmedizin Berlin Berlin Germany

**Keywords:** Bacille‐Calmette‐Guérin, immune response, infant, tuberculosis, vaccine research and development

## Abstract

**Aim:**

The tuberculosis (TB) vaccine, Bacille‐Calmette‐Guérin (BCG), protects infants against severe forms of primary TB. Yet, it does not protect against pulmonary reactivation TB in adults.

**Methods:**

A literature review was conducted between 1st January and 28th February 2025 using PubMed, Google Scholar and reports of the World Health Organization.

**Results:**

Protection against TB includes innate and acquired cellular immunity and infants are more susceptible due to immature immunity. Immunopathology of infant TB is characterised by formation of small granulomas in the lung often followed by dissemination to other organs. Infection in adults is frequently contained until immunity is weakened and TB disease develops. Novel vaccines are being developed mostly for adolescents and adults, but two live attenuated vaccines are currently assessed in infants in Phase III clinical trials. Globally, TB remains a major health threat and countries of the global south bear the major TB burden. Today, Sweden, similar to other high‐income countries, has a low incidence. General vaccination has therefore been terminated and targeted BCG vaccination is offered to infants at higher risk.

**Conclusion:**

Together with short‐treatment and rapid diagnosis, novel vaccines profoundly contribute to the fight against TB in all age groups.

**Abbreviations**
B: B lymphocyte
BCG: Bacille‐Calmette‐Guérin
EPI: Expanded Program for Immunisation
HIC: high‐income countries
IFN‐γ: Interferon‐γ
IGRA: Interferon‐γ Release Assay
IL‐17: Interleukin‐17

*L. monocytogenes*

:
*Listeria monocytogenes*

LMIC: low–middle‐income countries

*M. bovis*

:
*Mycobacterium bovis*

MDSC: myeloid‐derived suppressor cells
*Mtb*
:
*Mycobacterium tuberculosis*

PC: plasma cell
PoD: prevention of disease
PoI: prevention of infection
R&D: research and development
TB: Tuberculosis
Th1: CD4 T helper 1 lymphocyte
Th17: CD4 T helper 17 lymphocyte
Th2: CD4 T helper 2 lymphocyte
T_M_ cells: memory T cells
TNF‐α: Tumour necrosis factor α
T_reg_ cells: regulatory T cells
WHO: World Health Organization

**Summary**

- Tuberculosis remains a major health threat for all age groups and better vaccines are needed for improved global tuberculosis control.
- The current vaccine, Bacille‐Calmette‐Guérin, partially protects infants but not adults and therefore novel vaccine candidates primarily target adults although two candidates for infants are in late‐stage clinical development.
- Protective immunity is complex depending on a fine‐tuned balance between different T‐cell subsets which needs to be stimulated by vaccination.

## 1 Introduction

Tuberculosis (TB), caused by *Mycobacterium tuberculosis* (*Mtb*), is a devastating disease [1, 2]. Of the 10.8 million TB cases total, paediatric TB was responsible for about 12%–15% in 2023. Accordingly, 1.5 million infants and young children develop active disease annually [3]. At the same time, the TB vaccine, Bacille‐Calmette‐Guérin (BCG), is the most widely deployed childhood vaccine and some 200 million infants receive this vaccine every year [1, 4, 5]. BCG is part of the Expanded Program for Immunisation (EPI) which has been implemented in 1974. Over the last 50 years, an estimated 140+ million lives of children and infants has been saved by EPI vaccines with near to 11 million saved from TB death [6]. BCG protects against infant and childhood TB, notably against extrapulmonary forms including miliary TB and TB meningitis. In contrast, protection against pulmonary TB, notably in adolescents and adults is negligible [7, 8, 9]. With 1.25 million fatalities in 2023, *Mtb* remains the deadliest infectious agent on earth over the last centuries [1]. TB is also responsible for some 15% of all global deaths in children with the vast majority in the age group < 5 years [1, 3]. Thus, *Mtb* is also a major killer of infants and young children.

This dire fact led the World Health Organization (WHO) to declare TB a global health emergency already in 1990 when an estimated 3 million deaths occurred annually. Consequences were marginal, and WHO could never declare an end to the global health emergency for TB. In marked contrast, when WHO declared COVID‐19 a global health emergency in 2020, virtually unlimited funding for research and development (R&D) of efficacious vaccines was mobilised immediately. As a corollary, WHO could announce the end of the global health emergency for COVID‐19 in 2023 [7].

A first UN high‐level meeting on tuberculosis was convened in 2018. Activities, however, were overshadowed by the COVID‐19 crisis and hence a second UN high‐level meeting on tuberculosis took place, 22nd September 2023 [10]. The declaration included several highly ambitious targets for 2027 to 2030 including:

… at least 90% of the estimated number of people who develop tuberculosis are reached with quality assured diagnosis and treatment …. providing life‐saving treatment for up to 45 million people … including up to 4.5 million children and up to 1.5 million people with drug‐resistant tuberculosis …

… providing up to 45 million people with TB preventive treatment including 30 million household contacts of people with tuberculosis including children and 15 million people living with HIV…

The UN declaration was equally ambitious with regard to financial support by stating:

… mobilise sufficient, adequate, predictable and sustainable financing … global investments of at least USD 22 billion a year by 2027 …

… [mobilise] USD 5 billion a year by 2027, for the development of safe, effective, accessible and affordable, rapid and accurate point of care diagnostics, including for drug susceptibility testing, for use in community settings; vaccines for all forms of tuberculosis for people of all ages; and shorter, safer and more effective treatment regimens, …

Global TB control would benefit enormously from these ambitious goals, even though signatories are not accountable and the US administration has silenced several relevant commitments.

This treatise will describe: (i) our current knowledge about protective immunity against TB pointing out major differences between infants and adults; (ii) the current status of R&D for better vaccines against TB in all age groups with a particular emphasis on vaccines for paediatric TB; (iii) the status of BCG vaccination in Sweden; (iv) the need for, and impact of, future TB vaccines for global health. This discussion will be preceded by a brief history of BCG vaccination and its impact during the last century.

## 2 History of BCG

### 2.1 Attenuation of *Mycobacterium bovis*

At the time when BCG was developed, infant TB was often caused by *M. bovis* via contaminated milk [11]. With the advent of pasteurisation, the gastrointestinal route of infection has become rare and *Mtb* infection of infants typically occurs via the pulmonary system as for adults. BCG was named after its developers, Albert Calmette and Camille Guérin (Bacille‐Calmette‐Guérin) [11]. These researchers used ox bile mixed with glycerin to achieve fine dispersion of the bacilli. They continuously cultured *M. bovis* for 3 weeks and then transferred samples to a fresh culture medium. This process was repeated for 231 times over 13 years. Once bacteria had been found avirulent and protective against TB challenge in experimental animals as well as the natural host, cattle, the decision was made to assess the attenuated strain as a TB vaccine for human infants.

### 2.2 Clinical Testing of BCG

First clinical studies were performed by Weill‐Hallé and Turpin between 1921 and 1924, who immunised infants born into a household with at least one patient with active TB [12]. Evaluation of 20 000+ immunised neonates in 1927 revealed that only 1% of the immunised babies had died [11]. Although no placebo control had been included in these studies, it was generally assumed that, based on past epidemiologic observations, infant mortality was in the order of 5%–10%. Accordingly, BCG was highly protective. These data were later confirmed. A study performed in New York revealed that while mortality in neonates born from tuberculous mothers in hospitals was 8% in nonimmunised neonates, mortality was 0.9% in the immunised neonates during their first year of life [13]. Vaccination campaigns in several countries not only demonstrated specific protection against TB, but also reduced general mortality. Notably, in Sweden, infants immunised with BCG suffered from general mortality of 2.3%, whereas fatalities of nonimmunised infants was as high as 9.5%. These findings led Calmette to raise the question: [13]

… does the harboring of BCG … confer on the organism a special aptitude to resist those other infections which are so frequent in young children?

The heterologous resistance against other infectious diseases forms the basis for current discussions about heterologous effects conferred by BCG through trained innate immunity (see also chapter by Cai et al., in this volume) [14]. A recent reanalysis not only confirmed profound protection by BCG against TB, but also protection against all‐case mortality between 1927 and 1929 and against death by other respiratory infections between 1931 and 1932 [15].

### 2.3 The Lübeck Disaster

A major drawback to BCG vaccination campaigns occurred in 1930 when in the town of Lübeck in northern Germany 251 neonates had been vaccinated [16, 17]. Of these, 210 developed active TB, of whom 75 babies died from or with TB. 135 babies survived TB and 39 remained healthy from the beginning on (note, this was in preantibiotic times). Because the vaccine had been given per os, the gastrointestinal tract was particularly affected and, in several instances, primary pulmonary TB accompanied by extensive swelling and inflammation of mediastinal and tracheal lymph nodes was observed probably due to accidental aspiration. Miliary TB affecting various organs frequently occurred. At those times, the BCG vaccine was prepared at the hospital, where immunisation was performed with freshly made cultures from a seed lot provided by Calmette and Guérin. The accident was later demonstrated to be due to erroneous immunisation with a BCG culture contaminated with wildtype *M. tuberculosis* . No evidence was obtained directly linking BCG itself to the disaster [17].

### 2.4 Dose and Route of Immunisation

Originally, BCG was administered through the oral route at very high doses (1 billion (10^9^) bacilli suspended in milk) given several times. Subsequently, BCG was also applied subcutaneously which, however, caused local abscesses at high frequency. Ultimately, intradermal application of ca. 1 million (10^6^) bacilli turned out to be the administration of choice as discovered by Wallgren in the late 1920s [18]. With the formal adoption of intradermal vaccination, mass BCG campaigns gained momentum due to better vaccine uptake and standardisation providing higher consistency of vaccine batches and more streamlined rollout [19, 20, 21, 22].

### 2.5 Benefits and Failures of BCG

Several studies have analysed the benefits and failures of BCG in the past [9]. A recent meta‐analysis provided informative data on current BCG effectiveness [8]. As expected, BCG demonstrated significant protection against all forms of TB in children < 5 years (adjusted odds ratio 0.63). In addition, BCG also showed significant protection in children 5 to 9 years, given they had a positive tuberculin test or Interferon‐γ (IFN‐γ) Release Assay (IGRA) indicating ongoing *Mtb* infection (adjusted odds ratio 0.62). In contrast, participants of this age group, who had not experienced *Mtb* infection indicated by negative test, were not protected (adjusted odds ratio 1.29). Intriguingly, some protection against pulmonary TB was observed in children < 3 years (42% effectiveness) which contrasts several previous studies. In summary, the recent meta‐analysis further supports the notion that BCG vaccination causes effective protection against extrapulmonary TB in neonates and young children and provides some evidence for protection against pulmonary TB in the age group ≤ 3 years [8, 9]. Finally, this study suggests a longer lasting effectiveness of BCG in *Mtb*‐infected children 5 to 9 years. Therefore, apparently BCG loses its protective activity within 10 years at most.

## 3 Immunopathogenesis of Paediatric TB

Of the estimated 1.7 billion people infected with *Mtb*, ca. 10% will develop disease during lifetime [1]. Up to 5% develop active TB within the first 2 years after infection (Figure 1). Yet, others remain infected without any signs of active disease over decades [23, 24]. In contrast, a much higher proportion of infants and young children (up to 3 to 5 years) develop primary TB soon after infection. Between 5 and 10 years, risk of active TB is markedly reduced, but subsequently increases to reach a peak around 15 to 24 years of age which remains high over the next decades in males and slightly decreases in females (Figure 2) [1]. Neonates and young infants < 3 years are at highest risk of active TB because their immature immune response fails to control infection as efficiently as that of adults (see also chapters by Brodin and by Blom in this volume) [25, 26, 27, 28]. As a result, rapid progression to primary lung TB occurs followed by dissemination to other organs. On the other hand, disease is more often paucibacillary with a lowered risk of cavitation in infants [29, 30].

**FIGURE 1:**
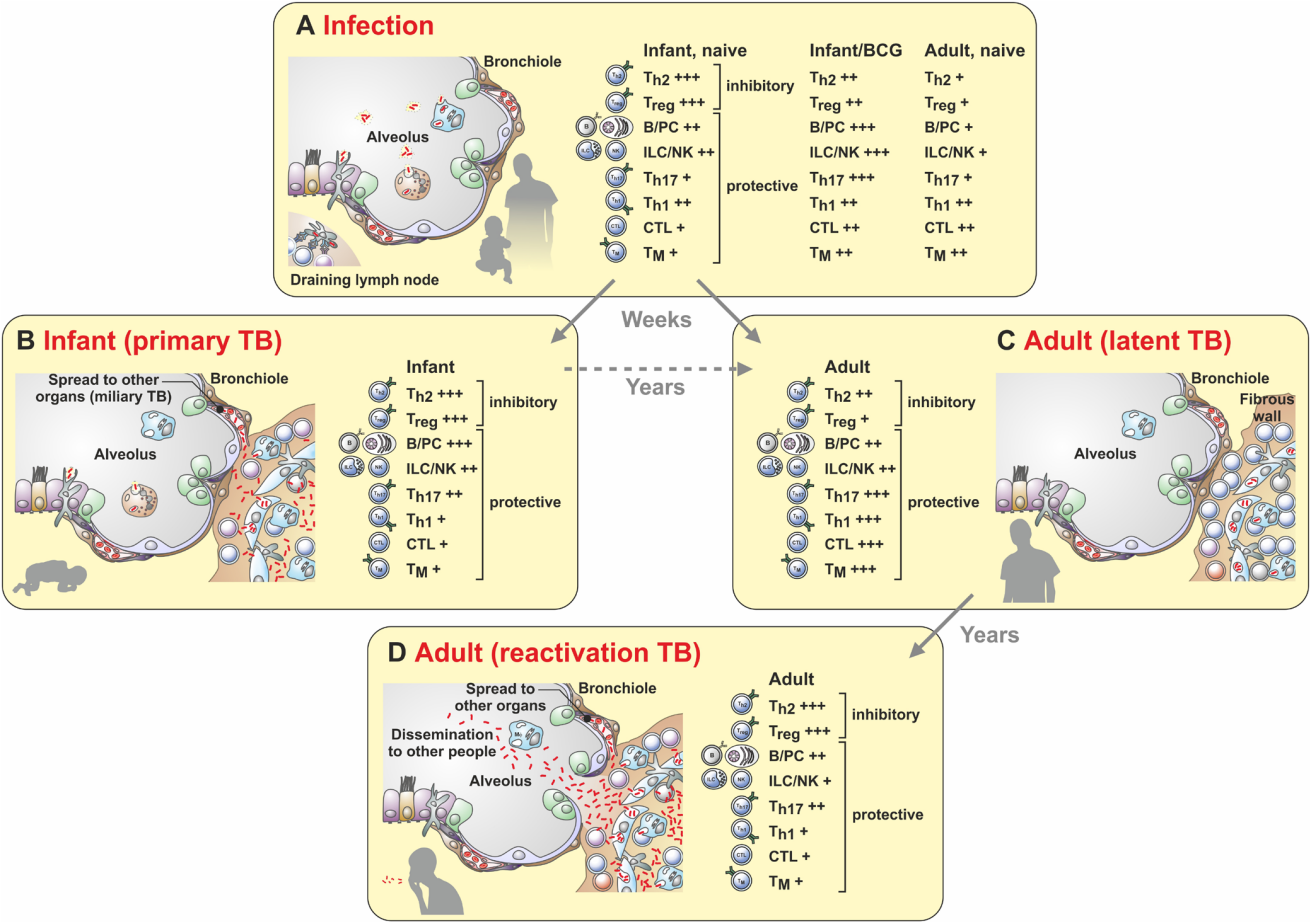
Hypothetical impact of different lymphocyte populations to tuberculosis (TB) and Bacille‐Calmette‐Guérin (BCG) in infants and adults. (A): Aerogenic *M. tuberculosis* infection and lymphocyte composition in infants with/without BCG and in adults. (B): Primary TB in infants and changes in lymphocyte composition. (C): Latent infection in adults and changes in lymphocyte composition. (D): Reactivation of TB in adults and changes in lymphocyte composition. Hypothetical alveolus granuloma settings in the lung as well as activity levels of major types of lymphocytes are depicted in each part. +, weak activity; ++, medium activity; +++, strong activity; B, B lymphocyte; CD4, CD4 T cells; CTL, cytolytic CD8 lymphocyte; ILC, innate lymphoid cell; NK, natural killer cell; PC, plasma cell; Th1, CD4 T helper 1 lymphocyte; Th17, CD4 T helper 17 lymphocyte; Th2, CD4 T helper 2 lymphocyte; T_M_, memory T lymphocyte; T_reg_, regulatory T lymphocyte.

**FIGURE 2:**
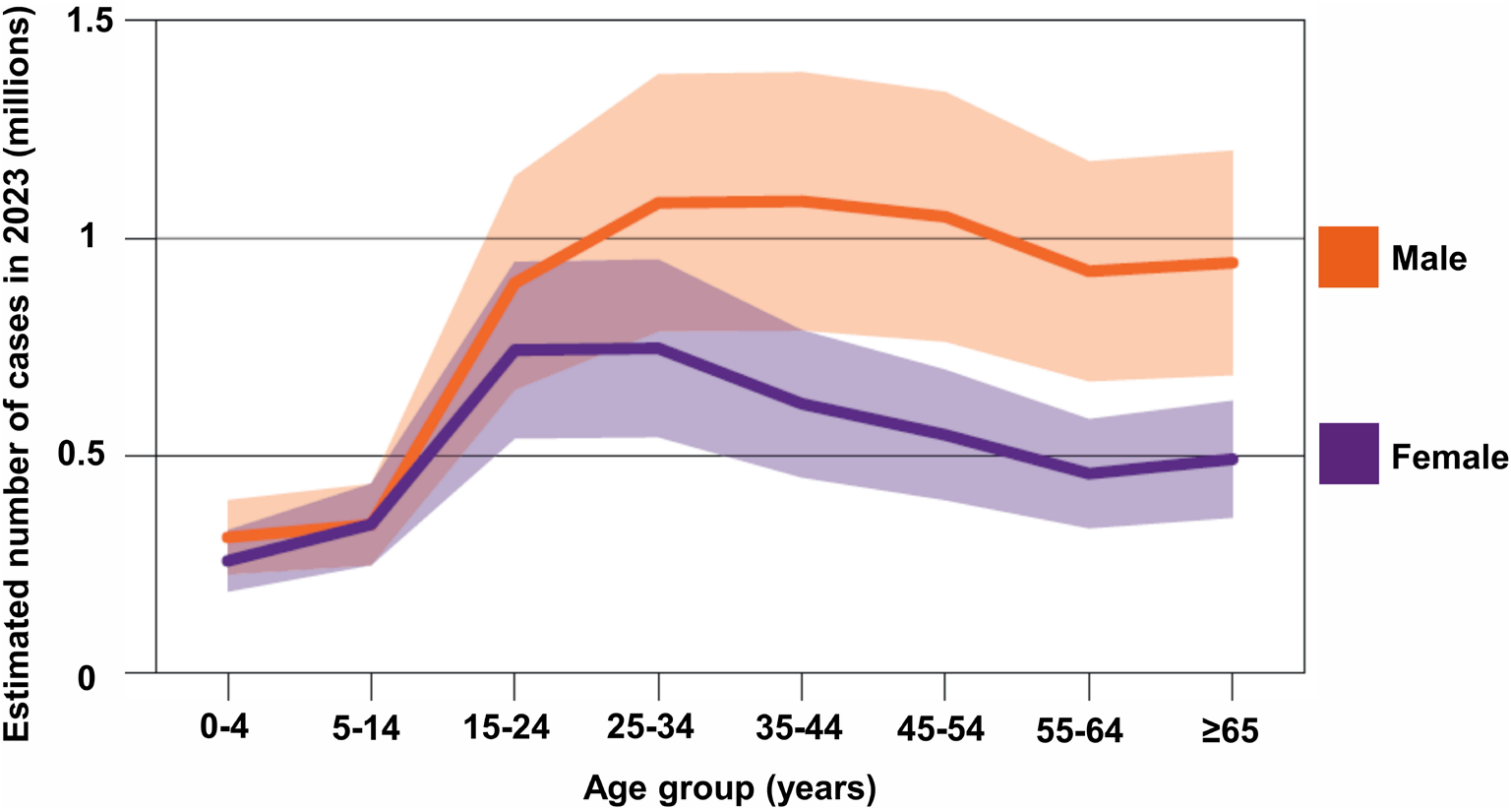
Age and sex distribution of TB cases. 55% of all TB cases are among men, 33% among women and 12% among children and young adolescents (< 15 years). The age group 0–4 years includes neonates (first month of life), infants (1 month—1 year), young children (1–5 years), elder children (5–14 years), paediatric cases (1–14 years), adolescents (15–19 years) and adults (> 20 years). Adapted from [1].

In the alveolar space phagocytes, both mononuclear phagocytes and polymorphic neutrophilic granulocytes represent the first line of defence [7, 24, 31]. Ultimately, *Mtb* organisms reach the draining lymph nodes, most likely via translocation by dendritic cells and mononuclear phagocytes. Here the acquired immune response is primed, leading to the activation of *Mtb*‐reactive CD4 and CD8 T lymphocytes, which subsequently migrate to the site of *Mtb* implantation, where they induce formation of granulomas which remain tiny and less structured but are more widespread over the affected organ than in adults [28, 29, 30]. Reduced activities of macrophages, neutrophils and dendritic cells may underlie insufficient containment of *Mtb* in these incomplete granulomatous lesions [28, 32]. Frequently, *Mtb* is disseminated to other organs to cause, for example, tuberculous meningitis, frequently complicated by severe neurological damage and hydrocephalus. In the worst‐case scenario, miliary TB develops characterised by tiny lesions of < 5 mm diameter in the numerous organs affected, such as lungs, spleen, liver, bone marrow and brain [28, 29, 30].

Obviously, lymphocytes are antigen inexperienced in infants and this could explain delayed immune responses towards BCG and possibly *Mtb* [33]. CD4 T cells of Th1 and Th17 types are considered critical for protective immunity against primary TB [7, 24, 31]. Accordingly, Type 1 and Type 17 cytokines including IFN‐γ, tumour necrosis factor α (TNF‐α) as well as interleukin‐17 (IL‐17) are relevant mediators of protective immunity (Figure 1). They do not directly attack *Mtb*. Rather, mononuclear phagocytes activated by Type 1 cytokines and fine‐tuned by costimulatory molecules are capacitated to kill or at least limit the growth of *Mtb*. Principally, infants generate a weaker Th1 response with lower IFN‐γ production, whereas Th17 cells are favoured in response to *Mtb* infection and BCG immunisation [25, 28]. CD8 T cells are regarded as critical as well. First, after *Mtb* infection and BCG immunisation, they produce Type 1 cytokines such as IFN‐γ and TNF‐α and hence can activate mononuclear phagocytes. Second, they have the capacity to lyse *Mtb*‐infected cells and to directly kill *Mtb* [34]. CD8 T‐cell activities are also weaker in infants [28, 35]. Impaired Th1 and CD8 T cell responses in infants are apparently compensated, at least in part, by stronger innate lymphoid cells including natural killer cells (Figure 1) [36]. Importantly, protective immunity depends on a fine‐tuned balance between different T‐cell populations including CD4 Th17 cells during the early stage and CD4 Th1 cells as well as CD8 T cells during subsequent stages (Figure 1).

B cells are stimulated in the draining lymph nodes in response to *Mtb* infection and BCG immunisation and subsequently develop into plasma cells producing *Mtb*‐specific antibodies [37, 38, 39]. In infants, maternal antibodies may contribute to humoral immunity [36]. While the role of T lymphocytes in immunity is beyond doubt, the contribution of antibodies and B cells remains a matter of discussion. It is likely that antibodies contribute to protection by improving defence mechanisms under the control of T lymphocytes.

On the other side of the equation, Th2 cells and regulatory T (T_reg_) cells keep the immune response under control, which is beneficial as long as protective mechanisms are favoured, but can become detrimental once immune reactions become hyperactivated and cause collateral damage. To prevent excessive inflammation, immunity in infants is biased towards Th2 and T_reg_ cells, most likely to maintain tolerance, for example, towards maternal antigens (Figure 1) [28]. Th2 cells induce antibody secretion by B cells and likely contribute to the healing of affected tissue sites after successful control of *Mtb* has been accomplished. Elevated immune control by T_reg_ cells in conjunction with myeloid‐derived suppressor cells (MDSC) has been found in infants and could contribute to their higher vulnerability for TB [40, 41].

Protection over prolonged times requires differentiation of T cells into memory T cells (T_M_ cells). Originally, T_M_ cells are subgrouped into central T_M_ cells and effector T_M_ cells [42]. More recently, interest has shifted towards resident T_M_ cells which are localised in the affected organ and therefore are well positioned for combating invading pathogens including *Mtb* [43, 44]. In infants and young children, T_M_ cell responses are weaker than in adults (Figure 1) [28, 36].

Taken together, infants as compared to adults show diminished activities of immune cells involved in protection against TB including macrophages, dendritic cells, Th1 cells and CD8 T cells as well as T_M_ cells and elevated activities of Th2 cells, T_reg_ cells and MDSC associated with immune inhibitory functions. Whilst it is most likely that the shift in the balance between protective and regulatory activities towards weaker effector functions and elevated regulatory functions is responsible for higher vulnerability of infants to TB and limited duration of BCG vaccine efficacy, the precise underlying mechanisms are far from being fully understood.

## 4 Lessons for Rational Vaccine Design

Our knowledge about vaccine‐induced protective immunity against TB remains insufficient although we increasingly understand the underlying mechanisms in more detail. A precise characterisation of how to best stimulate the most efficacious immune response defined as successful elimination of *Mtb* paralleled by low or minor collateral damage will be required for future rational design of next‐generation vaccines. BCG vaccination most likely focuses on stimulating CD4 Th1 and Th17 cells, but fails to adequately stimulate CD8 T cells [7, 24]. Apparently, the BCG‐induced immune response in infants is capable of preventing severe forms of primary TB, notably in the extrapulmonary environment. However, BCG seems to fail to induce long‐term immune protection capable of eradicating *Mtb* and hence cannot prevent reactivation of TB, notably in lungs later in life. Accordingly, induction of long‐term immune memory cells is a critical target of next‐generation vaccines against TB [45]. Since memory T‐cell responses are limited in infants and young children, booster vaccination between age 5 and 10 should be considered. Although the more ambitious approaches of TB vaccine R&D aim at prevention of stable infection (PoI) or eradication of ongoing infection for prevention of disease (PoD), a more modest approach targeting PoD by controlling ongoing latent infection over prolonged time periods could be a more realistic goal [7].

## 5 Novel Vaccine Candidates for Paediatric TB Vaccination

The virtual lack of protection by BCG against adolescent and adult TB has shifted the focus of R&D towards vaccines for the adult population. Principally, two TB vaccine types can be distinguished: first, whole cell vaccines; second, subunit vaccines [7]. A Phase IIb study of an early TB vaccine candidate—the viral vector Modified Vaccinia Ankara expressing Antigen 85A of *Mtb*—failed to show protection in infants [46]. Other subunit vaccines have undergone clinical trials for safety and immunogenicity, but were not further pursued in infants. Thus, only two live whole cell vaccines are currently in Phase III clinical trials in infants [7]. These are: MTBVAC (NCT 04975178), a genetically modified live *Mtb* vaccine and VPM1002 (NCT 04351685), a genetically modified live BCG. Both trials include HIV‐exposed/unexposed neonates (born by mothers living with/without HIV, respectively) to determine safety and/or protection in both populations as compared to BCG.

The MTBVAC vaccine was attenuated by genetic deletion of two independent virulence genes, PhoP and fadD26 [47]. The corresponding gene products regulate expression of some 100 genes and hence their deletion attenuates *Mtb* profoundly. Improved immunogenicity and safety of VPM1002 was accomplished by replacing the urease C gene by the gene encoding listeriolysin from *Listeria monocytogenes* [48]. This gene product is a cholesterol‐dependent pore‐forming cytolysin with a stringent need for an acidic pH. Listeriolysin promotes egression of *L. monocytogenes* into the cytosol, where it is rapidly degraded because it comprises a proline–glutamine–serine–threonine amino acid sequence that facilitates prompt degradation by the ubiquitin system [49]. To ensure the acidic pH optimum for listeriolysin expressed by BCG, ureaseC was deleted because BCG neutralises the phagosomal milieu. Accordingly, the vaccine candidate VPM1002, after phagocytosis by antigen‐presenting cells, secrets bioactive listeriolysin into the phagosome which perturbates the phagosomal membrane causing a number of intracellular alterations, resulting in higher immunogenicity and safety of VPM1002 over BCG [48].

Phase I and II studies have revealed safety and immunogenicity for both candidates, MTBVAC and VPM1002. Results from the Phase III vaccine trials in neonates are expected in 2026 for VPM1002 and a few years later for MTBVAC [7]. In addition, VPM1002 is being assessed in a Phase III trial for protection and safety against recurrence of TB in patients who had been cured by drug treatment but are at high risk of recurrence, either due to relapse or reinfection (NCT 03152903). Another Phase III clinical trial for safety and efficacy of VPM1002 in household contacts of patients with active TB has been completed (CTRI/2019/01/01706). Results of both studies are expected to be in the public domain soon. MTBVAC is currently in a Phase IIb trial in healthy infected adolescents and adults (NCT 06272812) and in a Phase I trial in adolescents and adults with/without HIV (NCT 05947890). Recently, the subunit vaccine, M72:AS01_E_, has entered Phase III clinical testing for safety and efficacy in adolescents and adults (NCT 06062238). In a Phase IIb trial, this vaccine had shown ca. 50% protective efficacy against TB disease in healthy infected individuals over 3 years [50].

Overall, the clinical trial pipeline of TB vaccines includes a dozen novel candidates and two novel immunisation schedules for BCG of adults [7]. With new data coming from vaccine trials in adults and new data from the two viable vaccine candidates in infants, opportunities are opening to evaluate promising candidates in prime‐boost combinations; in cluster‐randomised community trials; and in trials comprising longer lasting follow‐up over ≥ 5 years to cover the period of high susceptibility during the first year of life (40%–50% risk) and the more resistant stages from 5 to 15 years (5%–10% risk) (see Figure 2).

## 6 TB and BCG Vaccination Policies in Sweden

To illustrate the TB situation in high‐income countries (HIC), I chose Sweden as one of the first countries to introduce BCG vaccination (see 2.2). Around the turn of the 20th century, Sweden suffered from a high TB mortality rate of 200/100000. By 1927, when BCG was introduced in Sweden, incidences of TB had declined to 100/100000, still causing significant challenges [15, 19, 20, 21, 22]. In 1943, general BCG vaccination was officially adopted as public health intervention against TB. BCG coverage soon reached 95% and the vaccine was not only offered to newborn and young children, but also to school children 7 to 15 years of age, who were tuberculin test negative. Together with improved public health measures and better socioeconomic conditions as well as the introduction of antituberculous drugs, TB was gradually brought under control. Hence, in 1975, general BCG vaccination for infants was terminated in Sweden because the risk benefit ratio of adverse events vs. TB protection could be questioned at an incidence of 5/100000 cases.

In the 1980s/1990s, Sweden reported incidences of 4–6/100000 annually with > 70% of all cases in foreign‐born individuals with an incidence of 30–40/100000, whereas that of Swedish‐born infants was 1–2/100000 [21]. This led to the introduction of targeted BCG vaccination of risk populations accompanied by TB screening, contact tracing and free treatment for both latent and active TB in high‐risk groups. As a corollary, in Sweden TB incidences are at the lower level of TB rates similar to other HIC, but very different from numerous low–middle‐income countries (LMIC). While restricted BCG vaccination for high‐risk groups can be appropriate for HIC, general BCG vaccination needs to be maintained in LMICs.

## 7 Outlook

The global incidence rate of TB remains high at an average of 134/100000, with the majority of cases found in the 5 high‐burden countries comprising India (26%), Indonesia (10%), China (6.8%), The Philippines (6.8%) and Pakistan (6.3%) [1]. Although the majority of countries in sub‐Saharan Africa suffer from high TB incidence rates, they contribute less to the absolute number of TB cases because of their smaller population size than that in the major high‐burden countries. The situation is worsened by Rifampicin‐resistant (RR) and multidrug‐resistant (MDR, at least Rifampicin + Isoniazid‐resistant) accounting for some 420 000 cases in 2023, for whom treatment remains difficult to virtually impossible. Hence, the triad comprising rapid diagnosis, short‐term treatment and BCG vaccination remains high on the agenda to stop TB by 2027–2030 [51]. Novel diagnostic tests have been developed over recent years and are being introduced increasingly in high‐incidence countries [1]. Similarly, novel drug treatment regimens to shorten TB treatment have been developed [1]. BCG remains one of the most widely used vaccines for infants, with > 80% of 1‐year‐olds immunised. Yet, BCG does not protect against pulmonary TB in adolescents and adults and insufficiently in infants and young children (see 2.5). Indeed, novel TB vaccines are urgently needed for all age groups. Despite the generally assumed hesitancy of big pharma to invest in vaccine R&D for TB, which is most prevalent in LMICs, a significant market exists with up to 5.18 billion doses for adults as well as 1.4 billion doses for infants [4, 5]. This could translate into a substantial return on investment in the order of up to USD 475 billion or up to USD 100 billion averted for TB vaccines for adults or for infants, respectively.

## Author Contributions

S.H.E.K. conceived the idea and wrote the manuscript.

## Conflicts of Interest

S.H.E.K. is coinventor of the TB vaccine, VPM1002 and coholder of a patent licensed to Serum Life Science Europe GmbH (former Vakzine Projekt Management GmbH), Hannover, Germany and sublicensed to Serum Institute of India PVT. Ltd., Pune, India. The vaccine is currently undergoing Phase III efficacy trial testing.

## Data Availability

Data sharing not applicable to this article as no datasets were generated or analysed during the current study.
